# Requirements for the implementation of open door policies in acute psychiatry from a mental health professionals’ and patients’ view: a qualitative interview study

**DOI:** 10.1186/s12888-018-1866-9

**Published:** 2018-09-19

**Authors:** J. Kalagi, I. Otte, J. Vollmann, G. Juckel, J. Gather

**Affiliations:** 1 Department of Psychiatry, Psychotherapy and Preventive Medicine, LWL University Hospital, Ruhr University Bochum, Alexandrinenstr. 1-3, 44791 Bochum, Germany; 2 Institute for Medical Ethics and History of Medicine, Ruhr University Bochum, Markstr. 258a, 44799 Bochum, Germany

**Keywords:** Locked wards, Coercion, Open door policy, Acute psychiatry, Implementation, Qualitative research

## Abstract

**Background:**

Treating legally committed patients on open, instead of locked wards is controversially discussed and the affected stakeholders (patients, mental health professionals) have ambiguous views on the benefits and disadvantages. The study aims to assess the opinions and values of relevant stakeholders with regard to the requirements for implementing open wards in psychiatric hospitals.

**Methods:**

Semi-structured interviews were conducted with 15 psychiatrists, 15 psychiatric nurses and 15 patients, and were analyzed using qualitative content analysis.

**Results:**

The interviewees identified conceptual, personnel and spatial requirements necessary for an open door policy. Observation and door watch concepts are judged to be essential for open wards, and patients appreciate the therapeutic value they hold. However, nurses find the door watch problematic. All groups suggest seclusion or small locked divisions as a possible way of handling agitated patients. All stakeholders agree that such concepts can only succeed if sufficient, qualified staff is available. They also agree that freedom of movement is a key element in the management of acutely ill patients, which can be achieved with an open door policy. Finally, the interviewees suggested removing the door from direct view to prevent absconding.

**Conclusions:**

For psychiatric institutions seeking to implement (partially) open wards, the present results may have high practical relevance. The stakeholders’ suggestions also illustrate that fundamental clinical changes depend on resource investments which – at least at a certain point – might not be feasible for individual psychiatric institutions but presumably require initiatives on the level of mental health care providers or policy makers.

## Background

People suffering from severe and acute psychiatric disorders are at an increased risk of being involuntarily detained on a locked ward, especially in situations in which they pose a threat to themselves or to others. Although research by Rittmannsberger and colleagues [1], conducted in Austria, Hungary, Romania, Slovakia and Slovenia, showed more than 10 years ago that involuntarily committed patients are not necessarily referred to locked wards, it is still common practice in most European countries to treat acutely ill psychiatric patients who pose a danger to themselves or to others, at least initially on wards whose doors are permanently locked. Data from the United Kingdom even reveals that the proportion of locked wards has risen over the last decades, resulting in more than 90% locked wards out of all wards visited by the Care Quality Commission in 2015/2016 [2, 3].

In Germany, there are two legal regimes – the guardianship law (which is a federal law) and the mental health laws of each of the 16 German federal states – under which patients can be involuntarily admitted to a psychiatric hospital. About 10% of the admissions to a psychiatric hospital are involuntary, and the absolute number of involuntary admissions has increased significantly under both legal regimes since the 1990s [4]. Traditionally, legally committed patients are admitted to locked wards. However, in recent years, several clinicians and bodies advocated for stronger efforts to implement open door policies in psychiatry, which means to treat legally committed patients on open, rather than locked wards [5–8]. Such approaches are discussed controversially; however, some psychiatric hospitals have quite a long tradition of open door policies [9–11].

Legally, it is assumed that the treatment of legally committed patients on open, rather than locked wards, is permissible as long as hospitals take appropriate measures to guarantee that the respective patients do not abscond from the commitment [12, 13]. In North Rhine-Westphalia, which is the German federal state with the highest number of inhabitants, on 1 January 2017 a new mental health law came into force which even explicitly states that “legal commitments shall be performed in an open setting as far as possible” (Sec. 10 of the North Rhine-Westphalian PsychKG). All such demands and provisions aim to ensure the safety of the patients or others as best as possible while limiting the patients’ freedom as little as possible. There is data that opening the doors might lead to less aggressive incidents [14, 15] and that absconding and suicide rates are not higher compared with closed settings [15, 16].

However, previous studies revealed that mental health professionals’ and patients’ attitudes towards open wards are quite ambiguous and that different stakeholders tend to not only see benefits but also certain disadvantages of open wards, such as the loss of resources due to the observation of the open door or a decrease in security and control [2, 17–20]. Open door policies challenge those who are directly involved in clinical routines and have to transform theoretical concepts into daily clinical practice. While these previous studies have primarily investigated the effects of open door policies, there is no research that we are aware of that has explored how such concepts can be newly implemented and what organizational requirements may be necessary for putting them into practice.

The present study is part of a larger mixed-methods study on clinical effects and ethical aspects of open door policies. The mixed-methods study consists (1) of a prospective cohort study, which aims to investigate – amongst others – the effects of open compared to locked doors on coercive interventions and serious incidents in a sample of all patients who were involuntarily committed to five different psychiatric hospitals during a specified 6 months period, and (2) of a qualitative interview study with mental health professionals and patients. With the interviews, we aim to explore the experiences and attitudes of different experts of psychiatric practice with the implementation of open doors in an acute psychiatric hospital. In this article, we only present results of the qualitative interview study. We intend to identify requirements for the treatment of legally committed patients on open instead of locked wards by taking into account the practical and personal knowledge of those who are needed when such fundamental changes such as an open door policy are introduced into clinical practice.

## Methods

Between February and June 2016, four members of the research team conducted 45 qualitative, open-ended, semi-structured interviews (15 interviews with patients, 15 interviews with psychiatric nurses and 15 interviews with psychiatrists). Psychiatrists and psychiatric nurses were chosen because they are directly involved in the decision making around legal commitments of psychiatric patients and in putting those into practice (amongst others by determining whether the ward’s entrance door should be opened or locked). The patients were chosen because they are personally affected by the legal commitment and restricted in their freedom by the locked door.

Before starting the sampling process, the research project was presented to all psychiatrists and psychiatric nurses in their regular team meetings. The sampling process was purposive in order to obtain a diverse selection of psychiatrists and psychiatric nurses to represent a range of professional experience, hierarchical positions, ages and genders. The interviewees could get in touch or were contacted directly by the members of the research team. With regard to the patients, we contacted all patients who met the inclusion criteria and appeared in the hospital’s outpatient or inpatient department during the course of the study.

The inclusion criterion for psychiatrists and psychiatric nurses was to have working experience on both open and closed acute psychiatric wards. As for the patients, the inclusion criteria were (1) having a psychiatric disorder according to ICD-10, (2) having experiences with involuntary commitments in the hospital before and/or after the implementation of the open door policy and (3) preserved mental capacity at the time of the informed consent process and the interview. In case the patient had a legal guardian, the latter also had to give informed consent.

The study was approved by the ethics committee of the Medical Faculty of the Ruhr University Bochum (Reg. No. 15-5452). Before conducting the interviews, the interviewers were trained by an experienced sociologist with the help of mock interviews.

The semi-structured interviews focused on thematic aspects, such as personal experience with the open door policy, challenges and barriers as well as suggestions for improvement. The guideline was developed based on our literature review and refined further after the first interviews, giving the interviewers the opportunity to add relevant questions to the set. The average length of an interview was 41 min (range: 21–64 min). All interviews were audio-taped, anonymized and transcribed verbatim. All transcripts were read and reread to ensure familiarity with the data. The analysis was conducted by five members of the research team, who were not involved in the treatment of the patients. The coders followed the principles of qualitative content analysis, a method that provides useful access to the large amount of data by preserving the advantages of quantitative content analysis and complementing them with qualitative-interpretative steps of analysis [21, 22]. During the analysis, using AtlasTi 6.2 software, the data was repeatedly coded, moving from concrete passages to more abstract levels of coding, from codes to categories and finally to three overarching themes. This process was both inductively deriving themes from the data and searching for repeating concepts [23], as well as deductively analyzing the data on the grounds of previously conducted literature research and the current research question. These steps were repeated as coding guidelines for each interviewee group were gradually developed. All findings were critically tested and discussed among the researchers who had different disciplinary backgrounds (sociology, psychiatry, psychology, nursing and medical ethics). Any disagreements were resolved by discussion. Since the coding system remained the same for the last interviews and the findings did not add anything significantly new to the interviews conducted previously, we concluded that we had reached theoretical saturation.

As Table 1 demonstrates, the participants varied in their age and professional background or diagnosis respectively. One psychologist was included in the group of the psychiatrists. That particular psychologist carries out many tasks of the doctors in the clinic and is therefore referred to as part of the psychiatrist group for the remainder of this article.

**Table 1:** Clinical and socio-demographic characteristics

	Psychiatrists (*N* = 15)	Nurses (*N* = 15)	Patients (*N* = 15)	Total sample (*N* = 45)
Gender				
Female	9 (60%)	9 (60%)	3 (20%)	21 (47%)
Male	6 (40%)	6 (40%)	12 (80%)	24 (53%)
Age (M ± SD)	35.3 ± 7.0	35.2 ± 12.1	38.9 ± 14.0	36.5 ± 11.3
Range	28–54	24–63	20–60	20–63
Years of professional experience (M ± SD)	7.0 ± 7.0	12.0 ± 12.6		
Range	0.5–27	1.25–45		
Years employed in the hospital (M ± SD)	4.4 ± 6.0	6.7 ± 6.3		
Range	0.08–23.75	0.75–22		
Professional background				
Psychologist	1 (6.7%)			
Psychiatric resident	10 (66.7%)			
Psychiatrist (specialist)	2 (13.3%)			
Senior psychiatrist	2 (13.3%)			
Nursing student		2 (13.3%)		
Nurse without academic degree		10 (66.7%)		
Nurse with academic degree		3 (20%)		
Diagnosis				
Psychotic disorders (ICD-10 F2)			6 (40%)	
Affective disorders (ICD-10 F3)			6 (40%)	
Substance dependence (ICD-10 F1)			3 (20%)	
Duration of the illness in years (M ± SD)			17.0 ± 13.0	
Number of previous hospitalizations (M ± SD)			5.53 ± 6.3	
With legal commitment			2.00 ± 2.2	
Currently legally committed				
Yes			6 (40%)	
No			9 (60%)	

## Results

We identified three overarching themes in which changes are seen as necessary for the implementation of open door policy in an acute psychiatric hospital: conceptual, personnel and spatial requirements.

### Conceptual requirements

Two concepts which are often implemented in open door policies are intensive forms of observation and a door watch. The continuous or intermittent observation (e.g. 1:1 or 2:1 observation) of patients intends to ensure their safety and that of others and at the same time is a therapeutic intervention to engage the patient in a positive interaction. The door watch consists of a staff member being positioned near the door in order to prevent patients from absconding.

#### Observation

Both professional groups are in favour of observation because it is less disruptive of the patients’ freedom in comparison to a locked door or mechanical restraint. However, the nurses note that it is very exhausting to constantly be with a patient for lengthy periods of time. Furthermore, psychiatrists and nurses are aware that continuous observation may actually increase tension in certain patient groups. All interviewee groups acknowledge that observation is personnel intensive. Patients greatly appreciate continuous observation as it seems to achieve the right balance of feeling cared for while still remaining autonomous.

*I’ve had it a few times that a nurse offered me to talk a little or that I could reach out to them for a chat and I liked that very much. In general, that there are people around you and don’t harass you and don’t want anything but just have a look at what you’re doing, how you are. […] I’ve always found that pleasant.* (Patient 3)

#### Door watch

While psychiatrists and patients judge the door watch as largely positive, the nurses find the concept problematic. According to the nurses, the door watch poses a big challenge for several reasons. Firstly, they find it mentally draining to watch the door. Many experience stress because so much is happening in the entrance area and they have to be continuously alert. They report it is very difficult to make sure no patient absconds when they have to interact with other patients at the same time. They also experience stress because they are afraid of being attacked and getting harmed when a patient tries to abscond. Finally, some nurses report stress due to feeling responsible when a patient absconds and even being made responsible by their superior. The role of watching the door and ensuring that patients do not abscond thus comes with a lot of emotional distress.

*For a nurse, that’s not exactly a comfortable situation because you can’t assume that you can make the patient, who absolutely wants to leave the clinic now, a benevolent, therapeutic offer - rather, you have to mechanically prevent him from something. That’s always a negative experience to the point of quarrelling scenarios, which happen; with sounding the alarm and all that.* (Nurse 9)

Secondly, many nurses see a role conflict in being a “guard” for the door. Nurses view themselves as a caring profession whose primary concern is to support the patient. They are thus averse to the idea of having a task that puts them in a position of a guard. Moreover, they report that the door watch prevents them from being able to fulfill their role as a nurse. They can no longer assist patients because they cannot leave their spot or have therapeutic conversations with them because privacy is not given in the entrance area. For this reason, the nurses judge the door watch to be too resource intensive as an entire person’s capacity is taken up by a non-nursing task. Lastly, the nurses point out that repeatedly having to tell patients that they cannot leave, fosters aggression and sometimes strains the therapeutic relationship.

*You always had a bit of a bouncer feeling. You technically were rather a bouncer and not a nurse, but had to watch that he wouldn’t abscond, and this one, he can leave. Well that’s not really what we’ve studied for.* (Nurse 13)

Psychiatrists are in favour of having a nurse watch the door. They view a constant eye on the door as essential for the success of the open door concept. Psychiatrists think that when a patient leaves the ward, a fast reaction using the presence of numerous staff members is often very effective in preventing absconding. A prerequisite for this is a constant watch of the door.

*That would be, I’d say, a justifiable restriction of the respective person’s freedom. He would be brought back by nurses or an alarm would be sounded and everybody comes running. That usually is sufficient in getting the patient to return to the ward, and the other patients aren’t restricted one bit in their freedom of movement.* (Psychiatrist 9)

At the same time, psychiatrists acknowledge that nurses cannot be expected to verbally prevent all, especially aggressive patients, from absconding.

Patients experience a nurse paying attention to the door as largely positive. They appreciate that it gives them someone on the floor who they can talk to. Some patients feel like they are being closely monitored, which they dislike, while others mention that they do not have a feeling of being watched. Generally, patients think that by watching the door, nurses can keep a better eye on patients who are at risk of harming themselves or others which enables them to respond faster to escalating situations.

*Another one, two more would maybe be better because then maybe it would be a little more compact, right? Maybe one could leave the door open more often […] that staff sits there and pays special attention to those who are particularly at risk.* (Patient 12)

All groups agree that the majority of the patients can be easily convinced to stay on the ward by using verbal communication but that some patients cannot be reached by these means and have a strong urge to repeatedly try to leave the ward. The challenge of an open door policy is to find a way of managing this latter group of patients. The interviewees have various ideas of how this might be accomplished. All groups agree that a successful door watch requires more staff. Psychiatrists suggest that all wards should be built in a way that the door can be viewed from the nurses’ room. Another idea for managing challenging patients in an open setting involves seclusion.

#### Seclusion

Patients, nurses and psychiatrists unanimously would like to have the option of seclusion in the clinic. Patients reported finding seclusion less traumatic than mechanical restraint.

*Hence the rest room, the padded room. So they can go in there and let off steam without end and when they’re calm again, they can come out again; instead of being mechanically restrained. Because when you get mechanically restrained, it rather causes more frustration.* (Patient 1)

Professionals view a locked door as less coercive than mechanical restraint but they critically note that it affects many patients. By having a seclusion room, nurses highlight that a situation can be effectively de-escalated without having to mechanically restrain the patient or lock the ward for many unaffected patients.

*If the locked door can result in the de-escalation of a patient, or bring the situation as a whole under control, the locked door is preferable to mechanical restraint. But I would do it on a small scale […]. In my opinion, there should be an option to put the patient in a room, however that looks like, to lock the door and have a controlled area. I’d prefer that instead of having it affect so many patients.* (Nurse 11)

*For rather physically agitated and restless patients, if you can somehow create special options for them which lead to a reduction of this urge to move; well if you think beyond pharmacological stuff […] possibly a sort of rage room or soft room […] that aggression can also be released without injuries to themselves or others.* (Psychiatrist 12)

An overview of the identified conceptual requirements can be found in Table 2.

**Table 2:** Perceptions about conceptual requirements

	Psychiatrists	Nurses	Patients
Observation	+ less coercive than a closed door − long observation can create tension	+ less coercive than a closed door − long observation is exhausting	+ good balance between care and autonomy
Door Watch	+ effectively prevents most absconding − not all patients can be prevented from leaving	+ effectively prevents most absconding − very strenuous − safety risk − feeling too much responsibility	+ effectively prevents most absconding + conversation partner on the ward + better monitoring of dangerous patients − feeling of being watched
Seclusion	+ affects less patients than a closed ward	+ affects less patients than a closed ward	+ less traumatic than mechanical restraint

+: positive; −: negative

### Personnel requirements

#### More staff and strong therapeutic relationships

All interviewee groups agree that open-door policies can be implemented more successfully with more staff. Both professional groups critically note that the present staff number is insufficient when there are several acute patients on the ward or when staff members are ill. Especially continuous observation and the door watch, both of which are often carried out when the door is open, are very personnel-intensive and result in too little resources for the remaining patients. The patients are also aware of this issue.

*Worst-case-scenario would be being assigned to a shift with four of us. We have 32 patients who need to be cared for, then one of us drops out because of 1:1, then the door stays open so we need to establish a door watch, then the third nurse goes out on the floor. Then we only have two people who need to support the remaining 31 patients. That’s difficult.* (Nurse 8)

*The nurses are only humans, they can’t keep an eye on everything. They’ve also got paperwork, they also have to treat other patients and so the chance to abscond is easy. No that doesn’t work, it doesn’t work out.* (Patient 1)

Patients and nurses critically note that having too little time to engage in activities or conversations with patients gives patients the feeling that they are not being taken seriously. As a result, tension builds up. All three groups agree that building a strong relationship between staff and patient is crucial for an open door policy.

Psychiatrists and nurses suggest that by having more staff, even students, who can engage in continuous observation or watch the door, the other nurses have more time to manage the needs of patients and form stronger therapeutic relationships. Psychiatrists and nurses highlight that a strong relationship can prevent absconding because patients have a better understanding of why they are in the clinic and that staff want to help them. Moreover, the staff have a better understanding of how exactly they need to engage and communicate with the individual patient to calm them down or prevent them from leaving the ward.

*I for one had the feeling I often had too little time to engage in adequate relationship building with the patient, that I had the feeling relationship-wise things were running smoothly, so that patients on this basis could be prevented from absconding. And speaking from my medical point of view, I think there was too little of that. I would’ve preferred to talk more to the patients in order to clearly and explicitly discuss with them why they are here, so that on that basis of trust, I maybe would have accomplished more, that patients don’t abscond.* (Psychiatrist 3)

Patients and nurses suggest fixed opportunities for conversations with the staff. Patients report that it is important to them that they can talk to the nurses, to know that the staff regards them with benevolence and that the staff is open to make arrangements. Nurses stress that supporting patients, actively engaging and spending time with them can be effective in preventing tension from building up, which may have otherwise resulted in absconding or aggression.

*Beneficial here is without question the communication you can have here; that you’re not put off but rather that you can directly interact with the nursing staff, […] in the locked setting it’s rather that they withdraw, and with this observation there is a kind of care, you get attention and warmth and feel social proximity, and that’s very conducive to your health*. (Patient 2)

#### Trained staff

Nurses and psychiatrists emphasize that training staff adequately is very helpful in the implementation of open-door policies. Psychiatrists critically note that many staff members lack detailed knowledge on the legal aspects of coercive measures and legal commitments to a psychiatric hospital. Psychiatrists also admit that coercive measures, including the locked door policy, should be reviewed more regularly, because oftentimes short periods of a locked door are sufficient to de-escalate a situation. Psychiatrists suggest that there should be more standards and that the given standards regarding coercive measures should be more strictly adhered to. Nurses suggest regular supervisions or inter-professional reevaluation sessions in which the challenges and ideas for the implementation of the open door policy can be discussed.

*Technically, we are obliged to review every four hours, and also when a situation has de-escalated earlier, you should always check that you can quickly open the door again. And sometimes you become a little negligent with that.* (Psychiatrist 1)

An overview of the identified personnel requirements can be found in Table 3.

**Table 3:** Perceptions about personnel requirements

	Psychiatrists	Nurses	Patients
More staff	+ allows for observation and door watch while still having resources for unaffected patients	+ allows for observation and door watch while still having resources for unaffected patients	+ allows for an open ward with resources for all patients
Better therapeutic relationships	+ prevents absconding	+ prevents build-up of tension	+ assurance that staff regards them with benevolence
Trained staff	+ can decrease length of coercive measures	+ importance of re-evaluating situations	

+: positive

### Spatial requirements

#### Increased freedom of movement and outdoor activities

Both nurses and patients stress the importance of being surrounded by or in nature. They highlight that activities in nature have therapeutic and de-escalating effects. Patients would like to have more outside excursions with staff. Nurses express their wish to have more time to go for a walk with patients.

*That you get to do more with the group, go somewhere or go for a walk in the park, experience more nature, go for a jog or so.* (Patient 11)

Psychiatrists and nurses find a garden enclosed in the ward very valuable as patients can be outside, have a smoke and more space to move around. Patients appreciate it, too, but point out that the garden should not be too small.

*The problem was that I had to walk all the time because, due to the antipsychotics I got, I had restless legs symptoms. So somehow walk all the time, and so I constantly walked in a circle in this courtyard garden and was annoyed that I couldn’t get further out.* (Patient 3)

All three groups agree that it would be favourable to have a bigger space which patients can move in. However, psychiatrists note that a bigger space makes it hard to keep an overview and thus comes with safety risks.

#### No visible freedom restraints

Related to an increased freedom of movement is the suggestion of moving the actual border within which patients should remain. Both nurses and psychiatrists proposed that patients should be kept on the open ward through verbal communication and that there should only be a lock system outside the ward. This system may be an alarm that automatically goes off, a door further on in the clinic which automatically locks when an alarm button is pressed or special security staff who could be called.

*If you secure the outer borders, you could have plenty of latitude to possibly let the patient abscond because then you would find him on the way to the main reception so to speak. So really a mechanical process, that you kind of move the security aspect further outside distance-wise. To present the open here so to speak, which, I find and I stand by that, holds a therapeutic quality for the patient. But the security aspect, that no one absconds who’s going to run out in front of a car or who is going to kill someone or things like that.* (Nurse 9)

Another suggestion put forward by the nurses is the idea that it could be helpful to put the (open or locked) door out of line of sight for the patients. By moving the door around a corner which is not visible from the usual movement on the ward, the open door may not create such a big temptation to abscond while the locked door may not create so much frustration. Both these ideas attempt to make the freedom restraints less visible to the patient so that they are not constantly at the top of their minds.

#### Small locked divisions

Another compromise which is proposed by patients and nurses is to generally have an open setting in the clinic with an additional small locked ward or division. This locked area should have very few beds and only be for acutely dangerous patients. Furthermore, it should be highly staffed with experienced and trained personnel to ensure intensive care. Patients and nurses agree that such a ward would keep the other patients safe. The acute patients would also benefit because they could stabilize in a small, low-stimulus environment. Once they are stable again, they can return to their respective open ward.

*Maybe I would really stow them away in an extra space, well only at risk, strongly at risk […] that you can take them out in the acute phase but then also reintegrate them back into the community because they do belong to us, we are a community after all.* (Patient 15)

An overview of the identified spatial requiremenst can be found in Table 4.

**Table 4:** Perceptions about spatial requirements

	Psychiatrists	Nurses	Patients
Outdoor activities	+ therapeutic / de-escalating effects	+ therapeutic / de-escalating effects	+ therapeutic / de-escalating effects
Increased freedom of movement	+ de-escalating effects − safety risk	+ de-escalating effects	+ de-escalating effects
No visible restraints	+ compromise between perceived freedom and safety	+ compromise between perceived freedom and safety	
Small locked divisions		+ intense care for destabilized patients while maintaining an open setting	+ intense care for destabilized patients while maintaining an open setting

+: positive; −: negative

## Discussion

The interviewees identified several requirements for an implementation of open door policies which pose a challenge to the current mental health care system. These include – amongst others – door watch and special observation measures as conceptual requirements, a higher number of well trained staff as personnel requirements and more freedom of movement as well as making the door less visible as spatial requirements.

### Conceptual requirements

Special observation measures are well-established in many psychiatric institutions and an integral part of existing clinical guidelines [24]. Besides observational aspects, they entail therapeutic elements which are valuable for the management of suicidal or aggressive patients. If one opens a locked entrance door on a psychiatric ward, one has to replace the former “mechanical” with a “human” barrier to guarantee that the staff members always know who is on the ward and who is not. This requires measures with a strong focus on observation, and such measures may contradict the professional self-conception of nurses. Moreover, such observational measures can cause emotional distress among nurses (which was described as “anxious vigilance” by Muir-Cochrane et al. [19] several years ago) and come along with a feeling of having too much responsibility. Against this background, it seems desirable to 1. clearly assign responsibilities among the multi-professional teams, especially in situations in which it comes to absconding, 2. periodically change the observing nurse to prevent emotional distress and 3. strengthen the therapeutic elements in the implementation of a door watch which means using the time of the door watch procedure to engage patients in positive contact instead of merely focusing on the observational aspect.

The psychiatrists’, psychiatric nurses’ and patients’ call for seclusion seems to be quite contradictory at first sight, as it would merely replace one locked door (the ward’s entrance door) by another (the seclusion room’s door). Given that seclusion is rarely used in Germany, in comparison to other countries, and many German psychiatric hospitals do not even possess seclusion rooms (but use mechanical restraint instead [4]), this claim seems even more astonishing. Furthermore, the claim for seclusion apparently contradicts the international efforts to eliminate all coercive interventions [25–29] and seems to ignore the existing literature on negative effects of seclusion [30–32].

However, what concerns the psychiatrists and psychiatric nurses most is the management of individual agitated or aggressive patients without affecting many other patients by locking the door of the whole ward, and in this context, they regard seclusion rooms as helpful. These attitudes correspond – at least with respect to the patients’ and the staff members’ safety – to the results of a survey in which service users, carers and professionals to varying degrees (ranging from ca. 44 to 85%) hold the opinion that seclusion can increase the safety of service users or others [33].

The patients’ claim for seclusion rooms probably can be best understood in the context of their strong disapproval of mechanical restraint [34]. Patients might see seclusion as a less restrictive alternative compared to mechanical restraint [35].

In view of our results, alongside German guidelines which already recommend the provisioning of different types of coercive measures in psychiatric institutions (to be able to take into account the patients’ individual preferences [36]), psychiatric hospitals should consider providing seclusion rooms. This does not necessarily have to result in an increase of seclusion or coercive interventions in general. On the contrary, there is evidence that coercive interventions including seclusion can be significantly reduced by the introduction of an open-door policy [11, 14, 37]. Furthermore, there are less restrictive and voluntary alternatives to seclusion such as “soft rooms” which are already known from “Soteria” concepts [38]. Our interviewees suggested such facilities as helpful in the management of agitated patients.

### Personnel requirements

All stakeholders unanimously share the view that ambitious conceptual changes, like the implementation of an open door policy, can only succeed if sufficient and well qualified staff are made available. However, most of the legal reforms and various demands for a reduction of coercion in psychiatry are not accompanied by the provision of higher financial resources. Taking the views of those who are directly involved in daily clinical routines seriously, initiatives to reduce coercion should include sufficient financial and personnel investments in order to avoid excessive demands on the part of the staff and to make such initiatives successful. At the level of mental health care providers and leaders of psychiatric institutions, strong support should be provided by internal guidelines and regular trainings which has already been identified to be a helpful and effective approach in the reduction of coercion in general [39–42].

### Spatial requirements

Considering that legally committed patients are often tense and restless, an increased freedom of movement is presumably a key element in the management of patients in acute psychic crisis situations. Opening locked doors can both psychologically and factually increase the available space and help reduce crowding with all its negative effects on aggression and concomitant coercion [9, 15, 43–46].

With regard to the idea of moving the actual border out of sight of the patients, it is questionable whether an open door policy is still given when there is in fact a locked door further on in the building. However, the idea illustrates that professionals seek a compromise of reaping the therapeutic and ethical benefits of an open door while ensuring safety and abiding by the legal requirement of taking measures to keep the patient committed in the hospital.

Whereas such ideas would presumably require structural modifications in the hospital, this is all the more true for the idea of having small (locked) divisions for intensive care on a regular ward. The interviewees’ suggestions brings the Dutch “High and Intensive Care”- model to mind which entails special “Intensive Care Units”, in which patients can temporarily receive intensified care in acute crisis situations [47, 48].

All these approaches indicate that conceptual changes, at least at a certain point, often have to be accompanied by architectural changes. This is in line with the already existing evidence that modifying a hospital’s architecture can contribute to a reduction of coercion [49–52].

### Strengths and limitations

To our knowledge, this study is the first qualitative interview study on open door policies in acute psychiatry which includes psychiatrists, nurses and patients, hence all stakeholders who are primarily affected by such a far-reaching change in clinical practice. A key strength of this study is its use of a qualitative method to explore a multifaceted topic, enabling mental health professionals, alongside patients, to express their attitudes towards the implementation of an open door policy. Using a qualitative method such as semi-structures interviews allows participants to expand on their responses, ideally resulting in a rich data collection that provides depth and detail which could not be achieved using a quantitative method approach. However, selection biases due to the recruitment process are possible which could result in a bias towards the participation of mental health professionals who feel generally more positively towards this topic. Nevertheless, since we have (1) strictly respected confidentiality and anonymity and also (2) obtained a variety of distinct answers that are not limited to what would be expected to be socially desirable (e.g. very critical statements), it is safe to conclude that this bias remains small.

With regard to the participating patients, one major limitation is the fact that we did not systematically assess the patients’ disease severity at the time of the interviews and that some of their viewpoints might have been influenced by their current state of disease. Furthermore, not all patients we asked agreed to participate so that we possibly missed the views of those patients who would have been more critical.

A general limitation, which applies to most qualitative studies, is the issue of generalizability. However, the aim of qualitative studies is not generalizability or statistical significance, but rather, to gain a better understanding of social phenomena such as the effects of the implementation of open door policies. Since 1) the data was gathered from a good representation of various mental health professions and 2) theoretical saturation was reached within the number of conducted interviews, we are convinced that we have achieved this aim. Nevertheless, it is unclear whether the attitudes and beliefs presented here would be shared by participants from other settings in other areas or countries.

## Conclusion

Based on their personal experiences, mental health professionals and patients point out several requirements which help to promote the process of implementing open door policies in acute psychiatric hospitals. Hence, for all psychiatric institutions which seek to (partially) open former locked wards, their insights into conceptual, personnel and spatial preconditions might have a high practical relevance. On a broader level, the suggestions also illustrate that fundamental clinical changes, such as the implementation of open door policies, depend on resource investments which, at least at a certain point, might not be realised on the level of an individual psychiatric institution but presumably require initiatives on the level of mental health care providers or policy makers.

## Abbreviations

ICD-10: International statistical classification of diseases and related health problems (10th Revision)
PsychKG: Gesetz über Hilfen und Schutzmaßnahmen bei psychischen Krankheiten
